# Accuracy of adult height predictions in patients with axial leg deviations using the Modified and the Abbreviated Modified Fels Knee System

**DOI:** 10.1371/journal.pone.0311985

**Published:** 2024-11-12

**Authors:** Sebastian Braun, Niklas Thewes, Jana Holder, Marcus Rickert, Felix Stief, Marco Brenneis

**Affiliations:** 1 Department of Trauma Surgery and Orthopedics, University Hospital Frankfurt, Goethe University Frankfurt, Frankfurt, Main, Germany; 2 Center for Musculoskeletal Surgery, Charité ‐ Universitätsmedizin Berlin, corporate member of Freie Universität Berlin and Humboldt-Universität Zu Berlin, Berlin, Germany; 3 Department of Sport and Exercise Science, University of Salzburg, Hallein, Salzburg, Austria; 4 Berufsgenossenschaftliche Unfallklinik Frankfurt, Frankfurt, Main, Germany; University of Memphis, UNITED STATES OF AMERICA

## Abstract

**Background:**

The accurate estimation of residual growth is crucial for the appropriate timing of growth-guiding surgery in patients with axial leg deviations. Skeletal age methods such as the Modified and the Abbreviated Modified Fels Knee System were developed on historical patient cohorts and the applicability to the modern pediatric population with axial leg deviation has not yet been evaluated.

**Questions/purposes:**

1. Are both final adult height prediction methods (the Modified Fels Knee System (FKS) and the Abbreviated Modified Fels Knee System (aFKS)) accurate to determine SA and the final adult height on long leg radiographs in patients with axial leg deviations?
2. Which multiplier table shows highest association between predicted and true final adult body height?
3. Do FKS- and aFKS- skeletal age determination methods improve final adult body height prediction accuracy compared to the simple use of chronological age?

**Methods:**

A single center, retrospective study of 31 patients who underwent temporary hemiepiphysiodesis due to axial leg deviations in the frontal plane between 2018 and 2020 was conducted. Skeletal age at the time of surgery was determined on an anterior-posterior long leg X-ray using FKS and aFKS. Adult height predictions were calculated using three different multiplier tables (Paley et al., Sanders-Greulich and Pyle (SGP), Sanders-Peak Height Velocity (PHV)). The accuracy of adult height prediction was determined by comparing the mean differences and mean absolute differences between predicted and true adult height.

**Results:**

All adult height predictions overestimated the true adult height. The final height prediction using aFKS and the SGP multiplier showed the lowest overestimation (mean 3.2 cm, SD 5.5 cm). The PHV multiplier table showed the highest correlation between predicted and true adult height (FH_PRE_FKS_PHV_: r = 0.913, p < 0.001 and FH_PRE_aFKS_PHV_: r = 0.862, p < 0.001). The simple use of chronological age at the time of surgical intervention (CA_SI_) with the Paley multiplier table showed the highest median delta absolute values and lowest correlations with true adult height (median 7.4 cm, 25%-75% percentile: 3.5–10.0 cm, r = 0.838, p < 0.001). Nevertheless, no significant differences in delta absolute values between various adult height predictions methods could be shown.

**Conclusions:**

Overall, the results of the present retrospective cohort study show that there was no significant improvement in final height prediction accuracy when using the FKS or the aFKS method compared to the simple use of chronological age. One reason could be that patients with varus/valgus malalignment have specific growth characteristics that are not accounted for in multiplier tables or the FKS and aFKS method. Since there is no significant difference in prediction accuracy between the methods, the choice of method may depend on other factors, such as clinical preference or availability of resources. However, due to the small sample size, the study cannot definitively rule out potential differences between the prediction methods, and larger studies are required to validate these findings.

## Introduction

To optimize surgical treatment for axial leg deviations in the frontal plane (varus or valgus deformity), accurately determining the appropriate timing for growth-guiding surgery is crucial. Various methods are available for estimating residual growth and final adult height. Some methods utilize chronological age to determine the final adult height, while others rely on additional radiographs to ascertain skeletal age, subsequently using it to make predictions [1–5].

The determination of skeletal age commonly employs methods such as the Greulich and Pyle (GP) bone age atlas [5], the Sauvegrain method [4], and the Tanner-Whitehouse classification [3]. Hand-wrist radiographs are widely used for estimating skeletal age, with the GP atlas being a popular interpretation method. However, inaccuracies were identified in this method, leading to inconsistent skeletal age estimates among radiologists. In fact, 50% of the children had over 1 year difference in skeletal age estimations between different radiologists using this atlas [2]. To overcome these limitations, other methods such as the olecranon-only modification of the Sauvegrain method are recommended with higher intra-rater and inter-rater reliability compared to the GP atlas, providing a more consistent and precise approach to skeletal age estimation [1]. However, these evaluations are hindered by their subjective interpretation and the requirement for additional radiographs, such as those of the hand or elbow [6]. It is crucial to consider the increased radiation exposure, particularly for young patients, as the risk of radiation-induced malignancy correlates with the patient’s age [7]. To mitigate unnecessary radiation exposure, Benedick et al. [8] developed a method to determine skeletal age based on seven parameters of the knee. These seven parameters were selected from the original 36 parameters of the Roche-Wainer-Thissen method [9]. The Abbreviated Fels Knee System (aFKS) is a simplified version of the Modified Fels Knee System (FKS) [10]. In addition to the chronological age, only two to three further parameters are required. Thus, this method allows an even faster skeletal age determination compared to FKS. In terms of accuracy, it is said to be only slightly inferior to the FKS [10].

To compute the final adult height, additional calculations are necessary based on either the determined skeletal age or chronological age, such as using multiplier tables [11]. The most well-known multiplier tables were introduced by Paley et al. [11] and Sanders [12, 13]. These tables are frequently used in pediatric orthopedics to estimate final adult height using skeletal age or chronological age. Despite offering a convenient and rapid way to estimate final adult height, the accuracy of these multiplier tables has been challenged in recent years [14]. Moreover, the Sanders multiplier tables were developed using historical data from the Brush Foundation Study of Child Growth and Development, which primarily involved Caucasian, affluent, and healthy children. Consequently, their applicability to a modern, diverse patient population with axial deviations in the frontal plane may be limited [15, 16].

The aim of the current study was to evaluate the accuracy of adult height predictions in patients with axial leg deviations using the FKS and the aFKS with long leg AP radiographs and to compare the height predictions to the simple use of chronological age. We asked the following research questions:

1. Are both final adult height prediction methods (the FKS and the aFKS) accurate to determine skeletal age and the final adult height on long leg radiographs?
2. Which multiplier table shows highest association between predicted and true final adult body height?
3. Do FKS- and aFKS- skeletal age determination methods improve final adult body height prediction accuracy compared to the simple use of chronological age?

## Methods

### Patients

This single center, retrospective cohort study was approved by the institutional review board under the number 182/16. All investigations were performed in accordance with relevant guidelines and regulations. A prospectively maintained institutional database was used to identify 52 patients who were treated for varus or valgus deformity of the lower extremity. Patients were included if they had a preoperative long leg X-ray in an AP orientation and underwent temporary hemiepiphysiodesis between November 30^th^, 2018, and December 31^st^, 2020. The indication for implant-mediated growth guidance with hemiepiphysiodesis plating (Eight-Plates (Orthofix, Lewisville, TX, USA) or Pedi-Plates (Orthopediatrics Inc., Warsaw, IN, USA)) was set for skeletally immature patients with a pathological idiopathic valgus or varus alignment deformity (mechanical axis deviation of >10 mm and/or mechanical femorotibial angle of >3) of one or both lower extremities [17]. For this study, written informed consent was obtained from all participants involved. In the case of minor participants, written consent was additionally procured from their parents or legal guardians, in accordance with the ethical guidelines set by the institutional ethics committee and review board (IRB no. 182/16).

After surgery, patients were followed at regular intervals of 3–6 months until they reached adult height (at least until 1.5 years after implant removal (mean age 15.8 years (standard deviation (SD) 1.2)). The true final adult height (FH_TRUE_) was set as primary endpoint. FH_TRUE_ was determined when two conditions were met: the growth plates of the knee joint were closed, and there was no observed increase in body height over a twelve-month period. Height measurements were standardized and conducted in the orthopedic department, using the same calibrated stadiometer (wall-mounted height measuring device). All measurements were taken with shoes removed and with strict posture control to avoid flexion at the hip, knee, or ankle. Inadequate preoperative imaging with incomplete or rotated visualization of the area of interest (n = 2) or immaturity (implying patients’ incomplete growth and thus preventing final height determination) (N = 19) led to exclusion. Further exclusion criteria were: Knee surgery within 12 months before enrollment in this study, rheumatoid arthritis, neuromuscular disorders, achondroplasia or hypochondroplasia, sagittal plane deformities (genu pro- and recurvatum), flexion contractures in the hip or knee joint, leg length discrepancy of >10 mm, avascular necrosis of the femoral head or knee condyles or history of severe trauma or sport injury to the lower extremities. Ultimately, 31 patients (62 knees) were included within the study. Table 1 shows patients characteristics.

**Table 1:** Patient characteristics.

Patient Characteristics	
Patients, n	31
Sex, female: n (%) / male: n (%)	11 (35.5%) / 20 (64.5%)
Age_SI_total [years], mean (SD)	13.2 (1.1)
Age_SI_female [years], mean (SD)	11.9 (0.7)
Age_SI_male [years], mean (SD)	13.8 (0.6)
Age_LFU_total [years], mean (SD)	15.8 (1.2)
Age_LFU_female [years], mean (SD)	14.5 (0.7)
Age_LFU_male [years], mean (SD)	16.6 (0.7)

Age_SI–Age at surgical intervention (SI); Age_LFU–Age at 1.5 years after removal of implants (last follow up–LFU).

### Skeletal age evaluation with FKS and aFKS

Skeletal age at the time of surgical intervention (SA_SI_) was determined using the FKS and aFKS method [8, 10]. A scalable, standardized, digital long leg X-ray in an AP orientation was used to evaluate the respective SA_SI_ values. A 25.0 mm-diameter metal ball, which was placed between the legs at the level of the knee joint line was utilized to determine the individual magnification factor.

The FKS method is based on the evaluation of seven radiological parameters of the distal femoral and the proximal tibial growth plate: (1) Capping of the lateral distal femoral epiphysis over the metaphysis (FemK; 0 = absent, 1 = partial, 2 = complete); (2) Fusion of the lateral distal femoral physis (FemL; 0 = absent, 1 = partial, 2 = complete); (3) Capping of the lateral proximal tibial epiphysis over the metaphysis (TibN; 0 = absent, 1 = present); (4) Capping of the medial proximal tibial epiphysis over the metaphysis (TibP; 0 = absent, 1 = present); (5) Fusion of the lateral proximal tibial physis (TibQ; 0 = absent, 1 = partial, 2 = complete); (6) The ratio of proximal tibial epiphyseal width to metaphyseal width (TibA); (7) The ratio of proximal fibular epiphyseal width to metaphyseal width (FibA). In a next step, the initially evaluated parameters in combination with patients’ chronological age at surgical intervention (CA_SI_) and patients’ sex (female = 0; male = 1) were used to calculate the years from 90% of final height (Distance_90%FH_) by the following equation:

$${Distance}_{90\%FH}=0.379\left(CA_{SI}\right)-0.662(sex)+0.375(FemK)+0.237(FemL)+0.351(TibN)+0.289(TibP)+0.279(TibQ)+3.888(TibA)+2.395(FibA)-12.348$$ (1)

In a previously studied population, females reached 90% of final height at age 11.4 years and males at age 13.2 years [8]. Under this assumption, SA_SI_FKS_ was calculated using the following equations:

$$\text{Females:}\;SA_{SI-FKS}=11.4+{Distance}_{90\%FH}$$ (2)

$$\text{Males:}\;SA_{SI-FKS}=13.2+{Distance}_{90\%FH}$$ (3)

The aFKS method is based on the evaluation of five of the aforementioned radiological parameters of the distal femoral and the proximal tibial growth plate. Depending on the respective FemK-value SA_SI_aFKS_ was calculated by the following equations:

Females:

$$FemK-0:SA_{SI_{-}aFKS}=-5.842+0.526\left(CA_{SI}\right)+9.081(TibA)$$ (4)

$$FemK-1:SA_{SI_{-}aFKS}=-6.816+0.646\left(CA_{SI}\right)+9.222(TibA)$$ (5)

$$FemK-2:SA_{SI\_aFKS}=-7.188+0.408\left(CA_{SI}\right)+0.534(TibQ)$$ (6)

Males:

$$\left.FemK-0:SA_{SI_{-}aFKS}=-0.963+0.640\left(CA_{SI}\right)+4.344(TibA)\right)$$ (7)

$$FemK-1:SA_{S_{-}aFKS}=-5.640+0.628\left(CA_{SI}\right)+8.908(TibA)+0.466(\text{TibP})$$ (8)

$$FemK-2:SA_{S_{I}aFKS}=9.148+0.315\left(CA_{SI}\right)+0.653(TibP)+0.482(FemL)$$ (9)

Since all patients received therapy on both legs, the mean of the right and left skeletal age value was calculated and used for further analysis.

To ensure the reproducibility of the FKS and aFKS measurements, we conducted intra-rater reliability assessments as part of a separate, self-conducted study (unpublished data) on 98 data sets. Using a two-way mixed effects model with absolute agreement for single measures, we calculated Intraclass Correlation Coefficients (ICC). Observer A’s ICC for FKS was 0.938 (95% CI = 0.906–0.958), and Observer B’s ICC was 0.938 (95% CI = 0.859–0.968), indicating excellent reliability. For aFKS, ICC values were 0.842 (95% CI = 0.772–0.892) and 0.822 (95% CI = 0.667–0.897) for the two observers, demonstrating substantial reliability. These results affirm the consistency and reproducibility of the FKS and aFKS assessments.

### Multiplier tables

Next, three different multiplier tables were used to predict final adult height of the patients (FH_PRE_):

- Multiplier according to Paley et al. (2004) [11]: Paley et al. (P) used data from the Center for Disease Control and Prevention to create a multiplier table. Chronological age at surgical intervention (CA_SI_) and gender is needed to choose the respective multiplier value, which are given in monthly intervals. The final adult height of the patients (FH_PRE_P_) was calculated using the following equation:
$$FH_{PRE_{-}P}={Height}_{SI}*P\;multiplier\;value$$ (10)
- Multiplier according to Sanders–Greulich and Pyle (2017) [13]: Sanders et al. (SGP) used data from the Bolton Brush Study Foundation to evaluate skeletal age at surgical intervention (SA_SI_) and predict FH_pre_ by utilizing the GP atlas. Since both FKS and aFKS can determine skeletal ages that fall between the intervals of three months, a linear relationship between two neighboring multiplier values was assumed and thus the missing percentages were added (S1 Table). The final adult heights of the patients (FH_PRE_FKS_SGP_ and FH_PRE_aFKS_SGP_) were calculated using the following equation:
$$FH_{PRE_{-}SGP}={Height}_{SI}*\;SGP\;multiplier\;value$$ (11)
- Multiplier according to Sanders–peak height velocity (PHV) (2021) [12]: Sanders et al. (PHV) developed multiplier tables based on 90% of final height (90% FH). To use these multipliers for final adult height predictions, the distance between the average age at 90% FH (female: 11.4 years; male: 13.2 years) and the respective SA_SI_ was calculated and used to create the adapted multiplier tables (S1 Table). The final adult heights of the patients (FH_PRE_FKS_PHV_ and FH_PRE_aFKS_PHV_) were calculated using the following equation:
$$FH_{PRE_{-}PHV}={Height}_{SI}*\;PHV\;multiplier\;value$$ (12)

The use of CA and the different skeletal age prediction methods (FKS and aFKS) and the three multiplier tables (A-C) resulted in a total of nine predictions of FH_PRE_: (1) FH_PRE_CA_P_; (2) FH_PRE_CA_SGP_; (3) FH_PRE_CA_PHV_; (4) FH_PRE_FKS_P_; (5) FH_PRE_FKS_SGP_; (6) FH_PRE_FKS_PHV_; (7) FH_PRE_aFKS_P_; (8) FH_PRE_aFKS_SGP_; (9) FH_PRE_aFKS_PHV_.

### Statistical analysis

The accuracy of adult height prediction was determined by comparing the mean differences (*Delta* = *FH*_*PRE*_ − *FH*_*TRUE*_) and the mean absolute differences (*Delta Absolute* = |*FH*_*PRE*_ − *FH*_*TRUE*_|) between the respective FH_PRE_ and FH_TRUE_. The Shapiro-Wilk test was used to test normal distribution of the analyzed parameters. Continuous and normally distributed variables were presented as mean ± standard deviation (SD). Dependent and non-parametric variables were presented as median and interquartile range (IQR) and were compared between two groups using the Wilcoxon test. The calculated and normally distributed FH_PRE_ values were correlated to FH_TRUE_ using the Pearson correlation analysis (r). Multiple paired groups were compared with Friedman test. If the significance level for the Friedman test was less than .05 multiple comparisons were performed using the Dunn-Bonferroni test. A post hoc sensitivity power analysis was calculated in order to evaluate what effect sizes a within-subjects design is sensitive enough to detect. A Pearson correlation coefficient with 31 participants would be sensitive to effects of r = 0.59 with 95% power (alpha = .05, two-tailed). Sensitivity power analysis was performed using G*Power [18]. Statistical data analysis was performed with SPSS version 29 (IBM Corporation, New York, NY). The significance level was set at P ≤ 0.050.

## Results

### All FH_PRE_ overestimated FH_True_

**Fig 1:**
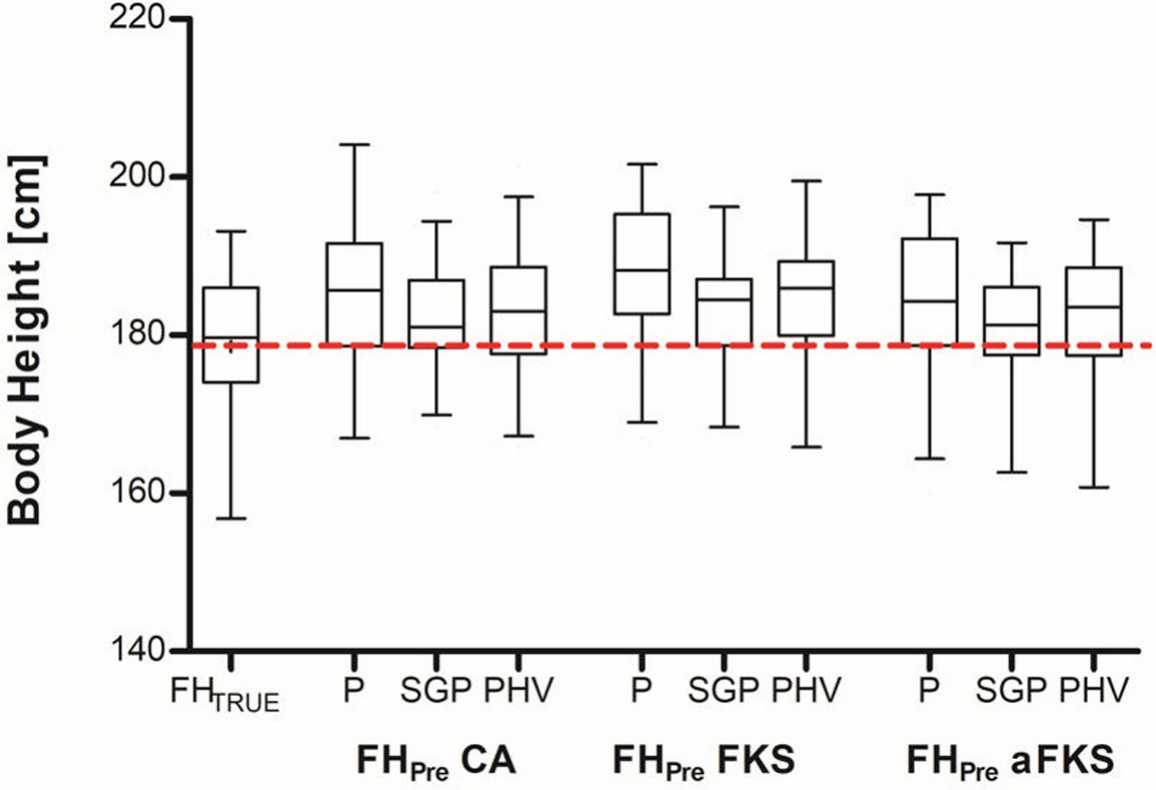
Overview of the calculated final adult height predictions compared to the true adult height (FH_True_). First, the chronological age at the time of surgery was used to predict the final adult height (FH_Pre_P_) using the Paley et al. (P) multiplier table, the multiplier according to Sanders–Greulich and Pyle (SGP), and the multiplier according to Sanders–peak height velocity (PHV). Second, skeletal age was determined using the Modified Fels Knee System (FKS) and the Abbreviated Modified Fels Knee System (aFKS) method. SA at the time of surgery was used to predict the final size (FH_Pre_) using two different multiplier tables (SGP and PHV). The dotted line shows the mean true adult height. Data represent medians with interquartile ranges. Whiskers represent Min to Max values.

The mean true adult body height (FH_True_) was 178.6 cm (min ‐ max: 156.8–193.2 cm). On average, all adult body height predictions overestimated the true adult body height (Fig 1). When comparing the deviation between predicted and true adult body height (*Delta* = *FH*_*PRE*_ − *FH*_*TRUE*_) FH_PRE_aFKS_SGP_ showed the lowest overestimation (mean 3.2 cm, SD 5.5 cm). In contrast, FH_PRE_P_ overestimated FH_TRUE_ by an average of 6.4 cm (SD 5.0 cm) (Table 2). In order to investigate which combination of multiplier and age determination method has the lowest deviation from FH_TRUE_, delta absolute values (*Delta Absolute* = |*FH*_*PRE*_ − *FH*_*TRUE*_|) were determined and compared in the following steps.

**Table 2:** Final height predictions.

	Mean [cm]	SD [cm]	Min [cm]	Max [cm]	Delta (FH_PRE_-FH_TRUE_) Mean [cm]	SD [cm]	Min [cm]	Max [cm]	Delta Absolut (FH_PRE_-FH_TRUE_) Median [cm]	25% percentile [cm]	75% percentile [cm]
Body Height_SI_	169.7	8.2	152.5	183.0							
FH_TRUE_	178.5	9.0	156.8	193.2							
**Chronological Age**											
FH_PRE_P_	185.0	8.3	167.0	204.1	6.4	5.0	-5.5	15.5	7.4	3.5	10.0
FH_PRE_SGP_	182.05	6.08	168.36	198.26	3.51	5.84	-10.76	15.14	4.71	1.44	9.66
FH_PRE_PHV_	182.73	7.58	165.92	201.37	4.19	5.14	-8.55	14.19	4.80	2.36	7.75
**FKS Mean**											
FH_PRE_FKS_P_	187.24	9.27	166.99	201.69	8.70	3.48	-0.79	15.75	8.99	7.29	11.11
FH_PRE_FKS_SGP_	**184.0**	**6.7**	**168.4**	**196.2**	**5.5**	**4.5**	**-6.3**	**15.2**	**5.4**	**2.7**	**9.9**
FH_PRE_FKS_PHV_	185.0	8.5	165.8	199.5	6.5	3.7	-3.4	14.9	6.8	4.6	9.0
**aFKS Mean**											
FH_PRE_aFKS_P_	184.47	8.40	160.51	197.71	5.93	4.35	-6.59	14,.2	6.32	4.29	9.11
FH_PRE_aFKS_SGP_	**181.7**	**6.3**	**162.6**	**191.7**	**3.2**	**5.5**	**-11.3**	**14.7**	**3.3**	**1.3**	**7.6**
FH_PRE_aFKS_PHV_	182.3	7.7	160.7	194.6	3.7	4.6	-9.5	13.4	3.9	2.3	7.0

FH_PRE_ ‐ Final adult height prediction; FH_TRUE_ ‐ True final adult height; SD ‐ standard deviation; FH_PRE_P_ ‐ Final adult height prediction using Payley multiplier; FH_PRE_SGP_ ‐ Final adult height prediction using Sanders ‐ Greulich and Pyle multiplier; FH_PRE_PHV_ ‐ Final adult height prediction using Sanders ‐ Peak Height Velocity multiplier; FKS ‐ Modified Fels Knee System; aFKS ‐ Abbreviated Fels Knee System; PHV ‐ Sanders ‐ Peak Height Velocity multiplier; SGP ‐ Sanders ‐ Greulich and Pyle multiplier; FH_PRE_FKS_P_ ‐ Final adult height prediction using FKS and Payley multiplier table; FH_PRE_FKS_SGP_ ‐ Final adult height prediction using FKS and SGP multiplier table; FH_PRE_FKS_PHV_ ‐ Final adult height prediction using FKS and PHV multiplier table; FH_PRE_aFKS_SGP_ ‐ Final adult height prediction using aFKS and SGP multiplier table; FH_PRE_aFKS_SGP_ ‐ Final adult height prediction using aFKS and SGP multiplier table; FH_PRE_aFKS_PHV_ ‐ Final adult height prediction using aFKS and PHV multiplier table.

### The PHV multiplier table showed highest association between predicted and true adult body height

**Fig 2:**
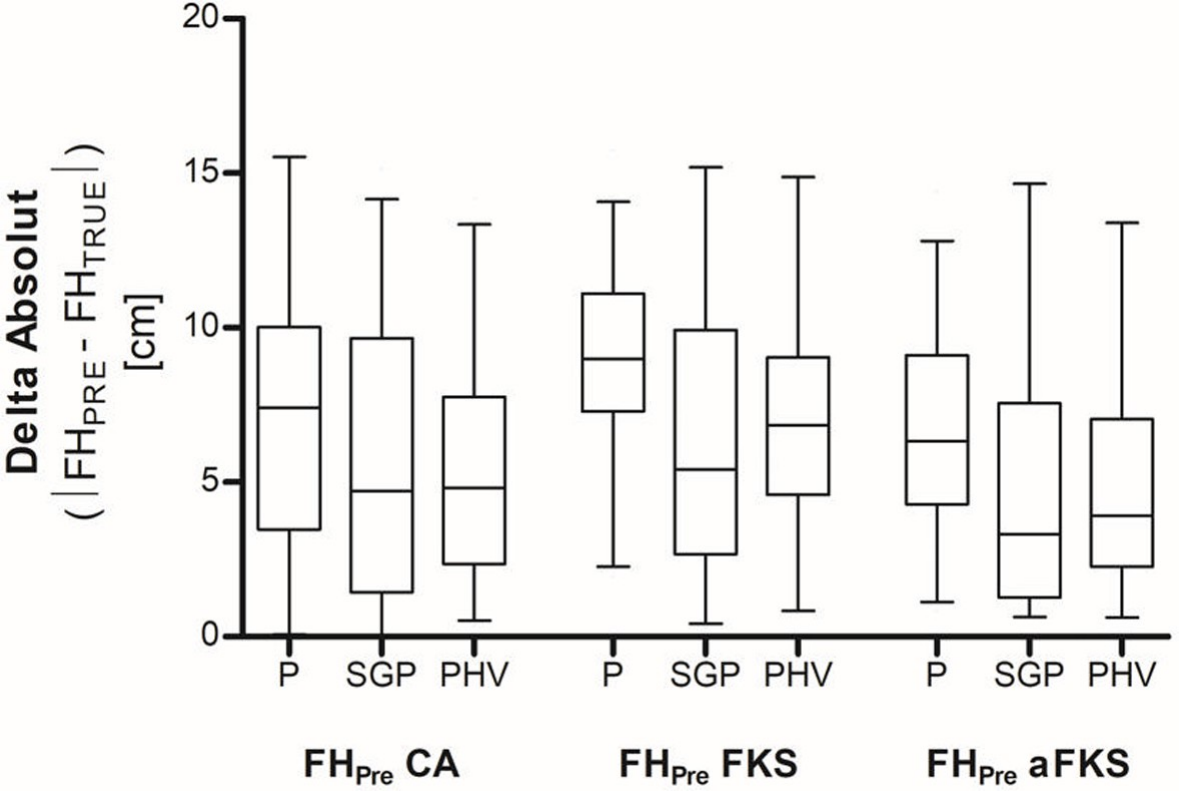
Final adult height prediction accuracy. The accuracy of adult height prediction was determined by comparing the mean absolute differences between the respective predicted final adult height (FH_PRE_) and the true final adult height (FH_TRUE_) (*Delta Absolute* = |*FH*_*PRE*_ − *FH*_*TRUE*_|). The chronological age at the time of surgery was used to predict the final adult height (FH_Pre_P_) using the Paley et al. (P) multiplier table, the multiplier according to Sanders–Greulich and Pyle (SGP), and the multiplier according to Sanders–peak height velocity (PHV). Skeletal age was determined using the Modified Fels Knee System (FKS) and the Abbreviated Modified Fels Knee System (aFKS) method. SA at the time of surgery was used to predict the final size (FH_Pre_) using two different multiplier tables (SGP) and PHV). Data represent medians with interquartile ranges. Whiskers represent Min to Max values.

When comparing the absolute deviation between predicted and true adult body height (*Delta Absolute* = |*FH*_*PRE*_ − *FH*_*TRUE*_|), FH_PRE_aFKS_SGP_ showed lowest median deviation (median 3.3 cm, 25%-75% percentile: 1.3–7.6 cm) (Fig 2). Although the SGP multiplier table showed lower median delta absolute values, the interquartile range of the PHV multiplier table was smaller in both FKS and aFKS. Nevertheless, we found no significant difference in delta absolute values between FH_PRE_FKS_SGP_ and FH_PRE_FKS_PHV_ (p = 1.0) as well as between FH_PRE_aFKS_SGP_ and FH_PRE_aFKS_PHV_ (p = 1.0). The simple use of CA_SI_ in combination with the Paley multiplier table (FH_PRE_P_) showed high median delta absolute values (median 7.4 cm, 25%-75% percentile: 3.5–10.0 cm). The use of skeletal age, evaluated by the FKS method in combination with the Paley multiplier table (FH_PRE_FKS_P_), showed the highest median delta absolute values (median 9.0 cm, 25%-75% percentile: 7.3–11.1 cm).

In addition, adult height predictions with the PHV multiplier table showed higher correlations with FH_TRUE_ than adult height predictions with the SGP multiplier table (FH_PRE_FKS_PHV_: r = 0.913, p < 0.001 vs. FH_PRE_FKS_SGP_: r = 0.874, p < 0.001 and FH_PRE_aFKS_PHV_: r = 0.862, p < 0.001 vs. FH_PRE_aFKS_SGP_: r = 0.803, p < 0.001). Adult height predictions with FH_PRE_CA_P_ showed lower correlations (r = 0.838, p < 0.001) with FH_TRUE_ (Table 3). In addition to the strong correlations observed for the FH_PRE_FKS_PHV_ and FH_PRE_aFKS_PHV_ predictions, the FH_PRE_FKS_P_ and FH_PRE_aFKS_P_ methods also demonstrated good correlations. However, it is important to note that the absolute deviations (Delta Absolute) between predicted and true adult height were higher in these methods, indicating that while the correlations were strong, the actual predictions deviated more from the true adult height in comparison to other methods.

**Table 3:** Correlation analysis between predicted and true final adult height.

	FH_TRUE_	
	Pearson Correlation (r)	Sig. (two-sided)
**FH**_**PRE_CA_P**_	0.838	<0.001
**FH**_**PRE_CA_SGP**_	0.769	<0.001
**FH**_**PRE_CA_PHV**_	0.822	<0.001
**FH**_**PRE_FKS_P**_	0.828	<0.001
**FH**_**PRE_FKS_SGP**_	0.874	<0.001
**FH**_**PRE_FKS_PHV**_	0.913	<0.001
**FH**_**PRE_aFKS_P**_	0.878	<0.001
**FH**_**PRE_aFKS_SGP**_	0.803	<0.001
**FH**_**PRE_aFKS_PHV**_	0.862	<0.001

FH_PRE_ ‐ Final adult height prediction; FH_TRUE_ ‐ True final adult height; FH_PRE_P_ ‐ Final adult height prediction using Payley multiplier; FKS ‐ Modified Fels Knee System; aFKS ‐ Abbreviated Fels Knee System; PHV ‐ Sanders ‐ Peak Height Velocity multiplier; SGP ‐ Sanders ‐ Greulich and Pyle multiplier; FH_PRE_CA_P_ ‐ Final adult height prediction using chronological age and Payley multiplier table; FH_PRE_CA_SGP_ ‐ Final adult height prediction using chronological age and SGP multiplier table; FH_PRE_CA_PHV_ ‐ Final adult height prediction using chronological age and PHV multiplier table; FH_PRE_FKS_P_ ‐ Final adult height prediction using FKS and Payley multiplier table; FH_PRE_FKS_SGP_ ‐ Final adult height prediction using FKS and SGP multiplier table; FH_PRE_FKS_PHV_ ‐ Final adult height prediction using FKS and PHV multiplier table; FH_PRE_aFKS_SGP_ ‐ Final adult height prediction using aFKS and Payley multiplier table; FH_PRE_aFKS_SGP_ ‐ Final adult height prediction using aFKS and SGP multiplier table; FH_PRE_aFKS_PHV_ ‐ Final adult height prediction using aFKS and PHV multiplier table.

### FKS and aFKS method did not significantly improve adult body height prediction accuracy

Adult height predictions with FH_PRE_CA_P_ showed lower correlations (r = 0.838, p < 0.001) with FH_TRUE_ than FH_PRE_FKS_PHV_ (r = 0.913, p < 0.001), FH_PRE_FKS_SGP_ (r = 0.874, p < 0.001), and FH_PRE_aFKS_PHV_ (r = 0.862, p <0 .001). When comparing all FH_PRE_ delta absolute values the Friedman test detected a significant difference (p < 0.001). Pairwise multiple comparisons using the Dunn-Bonferroni test showed significant differences in delta absolute values between FH_PRE_FKS_P_ and FH_PRE_aFKS_PHV_ (p < 0.001), FH_PRE_aFKS_SGP_ (p < 0.001), FH_PRE_CA_PHV_ (p < 0.001), FH_PRE_CA_SGP_ (p = 0.002) and FH_PRE_FKS_SGP_ (p < 0.017). However, additional pairwise comparisons using the Dunn-Bonferroni test did not detect any differences between the respective adult body height prediction methods (all p > 0.05).

## Discussion

This is the first study that examined the accuracy of adult height predictions in patients with axial leg deviations using the FKS and aFKS systems with long leg AP radiographs and compared these predictions to those obtained by simply using chronological age. The results showed that all tested final height prediction methods overestimated the true adult height (FH_True_). The PHV multiplier table exhibited the highest correlation between predicted and true adult height. FKS and aFKS did not significantly improve the accuracy of height predictions compared to the use of chronological age. The final height prediction using aFKS and the SGP multiplier showed the lowest overestimation of 3.2 cm and the smallest median delta absolute value of 3.3 cm. In contrast, the combination of skeletal age using the FKS method with the Paley multiplier table showed the worst estimation of true final height, with the highest mean overestimation of 8.7 cm and the largest median delta absolute value of 9.0 cm.

In this study, we concentrated on two methods for skeletal age determination and the evaluation of three specific multiplier tables (P [11], SGP [13], PHV [12]) for their effectiveness/accuracy in estimating FH_True_. Although the SGP multiplier table showed lower median delta absolute values, the interquartile range of the PHV multiplier table was smaller for both FKS and aFKS. Additionally, adult height predictions using the PHV multiplier table had higher correlations with FH_True_ than predictions using the SGP multiplier table. Therefore, the PHV multiplier table appears to be the more accurate. All adult height predictions tended to overestimate FH_True_ and it is unclear why even validated multiplier tables, like Paley et al. [11], significantly overestimated FH_True_. One reason for overestimation of FH_True_ could be that patients with varus/valgus malalignment have specific growth characteristics that are not accounted for in multiplier tables or the FKS and aFKS method. Another reason could be, that most multiplier tables and the FKS or aFKS method were developed on historical patient cohorts with different skeletal maturity patterns. In this context, Paley et al. [13] stated that height multipliers were consistent across generations, but their performance was unsatisfactory in this study, suggesting further inquiry and an updated method reflecting a diverse population with axial leg deviations. Growth-guiding interventions could result in a lower final height, and a study design with patients undergoing hemiepiphysiodesis on one side could test this hypothesis. Our study included patients affected and treated mainly on both sides.

The GP system with the use of hand/wrist X-ray is probably the most commonly used method for skeletal age determination. In order to prevent unnecessary radiation exposure, more recent methods such as the FKS or the aFKS are a useful alternative by providing options for skeletal age estimation in the management of pathologies of the lower extremity where long leg AP radiographs are acquired e.g., in the presence of axial deviations in the frontal plane. Nevertheless, these new sets of methods were developed using the same historic patient population of healthy individuals (Bolton-Brush Longitudinal Growth Inquiry). The FKS method, presented by Benedick et al. [8] in 2021, reported mean skeletal age prediction accuracy of ± 0.37 years and an interrater reliability of above 0.8 for each of the seven measured parameters [19]. The aFKS, introduced by Yuan et al. [10] in 2022, reduced the number of parameters to be measured from seven to two or three, making it faster to determine skeletal age. However, this also implies that an incorrect determination of one of the parameters would have a greater impact. Previous evaluations of some of these Bolton Brush-based methods for skeletal age determination have demonstrated that differences in performance can occur when applied to a heterogeneous patient population with modern clinical problems and underlying lower extremity pathologies [8, 20–22]. In this context, Furdock et al. [19] found differences between mean skeletal ages calculated using the FKS and actual chronological ages in a modern study population, calling into question the applicability of the method in a current study population. In the study by Furdock et al. [19], the skeletal age determined by the FKS method was on average 0.2 to 0.7 years higher than the chronological age. In the present study, the FKS method (r = 0.913 for PHV and r = 0.874 for SGP) also achieved slightly higher correlation values than the aFKS method (r = 0.862 for PHV and r = 0.803 for SGP). In this context, we have shown that using FKS to determine skeletal age with the use of PHV multiplier table results in the highest correlation between predicted and actual height. Thus, the correlations determined in the present study are consistent with the results of Furdock et al. [19]. They determined a correlation coefficient of r = 0.843 using the FKS method and the Paley multiplier table for the lower extremity. Nonetheless, it is important to note that different multiplier tables were used. Consistent with these findings, our results show a relatively high overestimation of FH_True_ when using FKS in a modern patient population (5.5 cm ± 4.5cm). This is why corrections for race, gender and other factors might be necessary to maintain the FKS applicability in modern pediatric populations. The results suggest that while the FKS method shows promising potential, adjustments to the formulas may be necessary to improve accuracy for modern patient populations, and future research should explore this possibility.

In the pursuit of a faster and more accurate method for skeletal age estimation, the aFKS with its reduced number of parameters has emerged as a promising alternative. Although this increases the impact of incorrect parameter determination, size predictions in the present study were more accurate overall with the aFKS than with the more comprehensive FKS method. Yuan et al. [23] stated that the mean difference to the actual skeletal age was ± 0.38 years for the aFKS method and ± 0.37 years for the FKS method. Our results failed to improve the prediction of height when using the FKS to estimate skeletal age and disagree with the findings of Furdock et al. [19] that the prediction of final adult height based on skeletal age by FKS was more accurate than that based on chronological age. While the application of the aFKS to full-length lower extremity radiographs did not yield perfect accuracy, our results nonetheless suggest that its performance in estimating skeletal age was not significantly inferior to that of the FKS.

In the treatment of axial deformity in the frontal plane, the FKS and aFKS methods may save patients additional radiation exposure compared to conventional methods of determining skeletal age that require additional radiography of the hand. Even though the results indicate that the FKS and aFKS methods did not significantly improve the accuracy of height predictions compared to the simple use of chronological age in combination with the Paley multiplier table, we recommend using the aFKS method due to the lowest absolute delta values and its fast implementation. Based on the results of this study, we do not recommend using the combination of skeletal age determined by the FKS method with the Paley multiplier table, as it resulted in the highest overestimation of true final height. However, the current findings should be considered as a tendency rather than a definitive conclusion. Further research, including larger sample sizes, is needed to determine the optimal method for predicting adult height in patients with axial leg deviations.

### Limitations

The results of this study need to be interpreted in the light of some limitations: First, this study was conducted with a sample size of 31 patients. Given the retrospective nature of our study, the sample size was constrained by the number of available records fitting the study criteria during the selected time frame. Although we evaluated a homogeneous group of children and adolescents with idiopathic knee valgus malalignment, the small sample size may limit the generalizability of the results. Second, it cannot be conclusively determined whether the study is transferable to a healthy patient population or a patient population with different pathologies because no control group was included in the study. However, the inclusion of a healthy control group would not have been ethically justifiable due to radiation exposure. Third, it cannot be assessed whether the correction of lower limb deformity in the frontal plane using hemiepiphyseodesis plating has an effect on the final adult body height and thus on the results of this study. Finally, we measured total body height as a surrogate for lower limb length, recognizing that this can change if the ratio of lower limb length to upper body length shifts during the final stages of growth.

## Conclusions

Overall, the results of the present study show that there was no significant improvement in final height prediction accuracy when using the FKS or the aFKS method compared to the simple use of chronological age. All methods overestimated the true final size. One reason could be that patients with varus/valgus malalignment have specific growth characteristics that are not accounted for in multiplier tables or the FKS and aFKS method. Therefore, an updated method reflecting a diverse population with axial leg deviations is necessary. Since there is no significant difference in prediction accuracy between the methods, the choice of method may depend on other factors, such as clinical preference or availability of resources.

## Supporting information

S1 Table Adjusted multiplier tables according to Sanders–Greulich and Pyle (SGP) and Sanders–peak height velocity (PHV). (DOCX)
