# Supplementary material for: Accuracy of adult height predictions in patients with axial leg deviations using the Modified and the Abbreviated Modified Fels Knee System
Source: PLoS One. 2024 Nov 12;19(11):e0311985. doi: 10.1371/journal.pone.0311985 (PMC11556750; doi:10.1371/journal.pone.0311985)
Supplement: S1 Table — (DOCX) [file pone.0311985.s001.docx]

**Suppl. Table 1** Adjusted multiplier tables according to Sanders – Greulich and Pyle (SGP) and Sanders – peak height velocity (PHV)

| **Male** | | |  | **Female** | | |  |
| --- | --- | --- | --- | --- | --- | --- | --- |
| **Years** | **Months** | **SGP-Multiplier** | **PHV-Multiplier** | **Years** | **Months** | **SGP-Multiplier** | **PHV-Multiplier** |
|  |  |  |  | 9 | 0 |  | 1,233 |
|  |  |  |  |  | 1 |  | 1,229 |
|  |  |  |  |  | 2 |  | 1,224 |
|  |  |  |  |  | 3 |  | 1,220 |
|  |  |  |  |  | 4 |  | 1,216 |
|  |  |  |  |  | 5 |  | 1,212 |
|  |  |  |  | 9,5 | 6 |  | 1,208 |
|  |  |  |  |  | 7 |  | 1,204 |
|  |  |  |  |  | 8 |  | 1,199 |
|  |  |  |  |  | 9 |  | 1,195 |
|  |  |  |  |  | 10 |  | 1,191 |
|  |  |  |  |  | 11 |  | 1,187 |
|  |  |  |  | 10 | 0 | 1,179 | 1,183 |
|  |  |  |  |  | 1 | 1,175 | 1,179 |
|  |  |  |  |  | 2 | 1,171 | 1,174 |
|  |  |  |  |  | 3 | 1,167 | 1,170 |
|  |  |  |  |  | 4 | 1,163 | 1,166 |
|  |  |  |  |  | 5 | 1,159 | 1,162 |
|  |  |  |  | 10,5 | 6 | 1,155 | 1,157 |
|  |  |  |  |  | 7 | 1,151 | 1,153 |
|  |  |  |  |  | 8 | 1,147 | 1,148 |
|  |  |  |  |  | 9 | 1,143 | 1,144 |
|  |  |  |  |  | 10 | 1,139 | 1,139 |
|  |  |  |  |  | 11 | 1,135 | 1,134 |
| 11 | 0 | 1,211 | 1,215 | 11 | 0 | 1,131 | 1,130 |
|  | 1 | 1,207 | 1,212 |  | 1 | 1,128 | 1,125 |
|  | 2 | 1,204 | 1,208 |  | 2 | 1,125 | 1,120 |
|  | 3 | 1,200 | 1,205 |  | 3 | 1,122 | 1,116 |
|  | 4 | 1,196 | 1,202 |  | 4 | 1,119 | 1,111 |
|  | 5 | 1,193 | 1,200 |  | 5 | 1,116 | 1,106 |
| 11,5 | 6 | 1,189 | 1,197 | 11,5 | 6 | 1,113 | 1,102 |
|  | 7 | 1,185 | 1,194 |  | 7 | 1,11 | 1,097 |
|  | 8 | 1,180 | 1,191 |  | 8 | 1,107 | 1,092 |
|  | 9 | 1,176 | 1,187 |  | 9 | 1,104 | 1,088 |
|  | 10 | 1,171 | 1,183 |  | 10 | 1,101 | 1,083 |
|  | 11 | 1,167 | 1,179 |  | 11 | 1,098 | 1,079 |
| 12 | 0 | 1,163 | 1,174 | 12 | 0 | 1,095 | 1,075 |
|  | 1 | 1,158 | 1,170 |  | 1 | 1,091 | 1,071 |
|  | 2 | 1,154 | 1,166 |  | 2 | 1,088 | 1,066 |
|  | 3 | 1,149 | 1,162 |  | 3 | 1,084 | 1,062 |
|  | 4 | 1,145 | 1,157 |  | 4 | 1,081 | 1,058 |
|  | 5 | 1,140 | 1,153 |  | 5 | 1,077 | 1,055 |
| 12,5 | 6 | 1,136 | 1,149 | 12,5 | 6 | 1,074 | 1,052 |
|  | 7 | 1,131 | 1,144 |  | 7 | 1,070 | 1,049 |
|  | 8 | 1,126 | 1,140 |  | 8 | 1,066 | 1,045 |
|  | 9 | 1,121 | 1,135 |  | 9 | 1,063 | 1,042 |
|  | 10 | 1,115 | 1,130 |  | 10 | 1,059 | 1,039 |
|  | 11 | 1,110 | 1,126 |  | 11 | 1,056 | 1,037 |
| 13 | 0 | 1,105 | 1,121 | 13 | 0 | 1,052 | 1,035 |
|  | 1 | 1,100 | 1,116 |  | 1 | 1,048 | 1,033 |
|  | 2 | 1,095 | 1,111 |  | 2 | 1,045 | 1,031 |
|  | 3 | 1,090 | 1,106 |  | 3 | 1,041 | 1,029 |
|  | 4 | 1,084 | 1,101 |  | 4 | 1,037 | 1,027 |
|  | 5 | 1,079 | 1,096 |  | 5 | 1,034 | 1,026 |
| 13,5 | 6 | 1,074 | 1,091 | 13,5 | 6 | 1,030 | 1,024 |
|  | 7 | 1,070 | 1,086 |  | 7 | 1,027 | 1,023 |
|  | 8 | 1,065 | 1,081 |  | 8 | 1,025 | 1,022 |
|  | 9 | 1,061 | 1,077 |  | 9 | 1,022 | 1,020 |
|  | 10 | 1,057 | 1,072 |  | 10 | 1,019 | 1,019 |
|  | 11 | 1,052 | 1,068 |  | 11 | 1,017 | 1,018 |
| 14 | 0 | 1,048 | 1,064 | 14 | 0 | 1,014 | 1,017 |
|  | 1 | 1,046 | 1,059 |  | 1 | 1,013 | 1,016 |
|  | 2 | 1,044 | 1,055 |  | 2 | 1,013 | 1,015 |
|  | 3 | 1,042 | 1,052 |  | 3 | 1,012 | 1,014 |
|  | 4 | 1,040 | 1,050 |  | 4 | 1,012 | 1,013 |
|  | 5 | 1,038 | 1,047 |  | 5 | 1,011 | 1,012 |
| 14,5 | 6 | 1,036 | 1,044 | 14,5 | 6 | 1,011 | 1,012 |
|  | 7 | 1,034 | 1,042 |  | 7 | 1,010 | 1,011 |
|  | 8 | 1,032 | 1,039 |  | 8 | 1,009 | 1,010 |
|  | 9 | 1,030 | 1,036 |  | 9 | 1,009 | 1,010 |
|  | 10 | 1,028 | 1,034 |  | 10 | 1,008 | 1,009 |
|  | 11 | 1,026 | 1,031 |  | 11 | 1,008 | 1,009 |
| 15 | 0 | 1,024 | 1,028 | 15 | 0 | 1,007 | 1,008 |
|  | 1 | 1,022 | 1,026 |  | 1 | 1,007 |  |
|  | 2 | 1,021 | 1,023 |  | 2 | 1,006 |  |
|  | 3 | 1,019 | 1,022 |  | 3 | 1,006 |  |
|  | 4 | 1,017 | 1,020 |  | 4 | 1,005 |  |
|  | 5 | 1,016 | 1,019 |  | 5 | 1,005 |  |
| 15,5 | 6 | 1,014 | 1,018 | 15,5 | 6 | 1,004 |  |
|  | 7 | 1,013 | 1,016 |  | 7 | 1,004 |  |
|  | 8 | 1,012 | 1,015 |  | 8 | 1,003 |  |
|  | 9 | 1,011 | 1,014 |  | 9 | 1,003 |  |
|  | 10 | 1,009 | 1,013 |  | 10 | 1,002 |  |
|  | 11 | 1,008 | 1,013 |  | 11 | 1,002 |  |
| 16 | 0 | 1,007 | 1,012 | 16 | 0 | 1,001 |  |
|  | 1 | 1,007 | 1,011 |  |  |  |  |
|  | 2 | 1,006 | 1,010 |  |  |  |  |
|  | 3 | 1,006 | 1,010 |  |  |  |  |
|  | 4 | 1,006 | 1,009 |  |  |  |  |
|  | 5 | 1,005 | 1,009 |  |  |  |  |
| 16,5 | 6 | 1,005 | 1,008 |  |  |  |  |
|  | 7 | 1,005 | 1,008 |  |  |  |  |
|  | 8 | 1,004 | 1,007 |  |  |  |  |
|  | 9 | 1,004 | 1,007 |  |  |  |  |
|  | 10 | 1,004 | 1,006 |  |  |  |  |
|  | 11 | 1,003 | 1,006 |  |  |  |  |
| 17 | 0 | 1,003 | 1,005 |  |  |  |  |
